# The Indirect Effect of Trauma via Cognitive Biases and Self-Disturbances on Psychotic-Like Experiences

**DOI:** 10.3389/fpsyt.2021.611069

**Published:** 2021-03-29

**Authors:** Renata Pionke-Ubych, Dorota Frydecka, Andrzej Cechnicki, Barnaby Nelson, Łukasz Gawęda

**Affiliations:** ^1^Experimental Psychopathology Lab, Institute of Psychology, Polish Academy of Sciences, Warsaw, Poland; ^2^Department of Psychiatry, Wroclaw Medical University, Wroclaw, Poland; ^3^Department of Community Psychiatry, Chair of Psychiatry, Medical College Jagiellonian University, Krakow, Poland; ^4^Orygen, Parkville, VIC, Australia; ^5^Centre for Youth Mental Health, The University of Melbourne, Parkville, VIC, Australia; ^6^Medical University of Warsaw, Warsaw, Poland

**Keywords:** self-disturbances, psychotic-like experiences, adverse life events, trauma, cognitive biases, psychosis risk

## Abstract

Although self-disturbances (SD) are considered to be a core psychopathological feature of schizophrenia spectrum disorders, there is still insufficient empirical data on the mechanisms underlying these anomalous self-experiences. The aim of the present study was to test a hypothesized model in which cognitive biases and exposure to traumatic life events are related to the frequency of SD which, in turn, contribute to the frequency of psychotic-like experiences (PLEs). Our sample consisted of 193 Polish young adults from the general population (111 females; 18–35 years of age, M = 25.36, SD = 4.69) who experience frequent PLEs. Participants were interviewed for PLEs, SD and social functioning as well as completed self-reported questionnaires and behavioral tasks that measure cognitive biases (e.g., safety behaviors, attention to threat, external attribution, jumping to conclusion, source monitoring, overperceptualization). The model was tested using path analysis with structural equation modeling. All of the hypothesized relationships were statistically significant and our model fit the data well [χ^2^(23) = 31.201; *p* = 0.118; RMSEA = 0.043 (90% CI = 0.00–0.078), CFI = 0.985, SRMR = 0.041, TLI = 0.976]. The results revealed a significant indirect effect of traumatic life events on PLEs through SD and self-reported cognitive biases. However, performance-based cognitive biases measured with three behavioral tasks were unrelated to SD and PLEs. The frequency of SD explained a substantial part (43.1%) of the variance in PLEs. Further studies with longitudinal designs and clinical samples are required to verify the predictive value of the model.

## Introduction

Phenomenological analyses along with empirical studies suggest that self-disturbances (SD), which are anomalous experiences of basic sense of self, are the core psychopathological feature and phenotypic trait marker of schizophrenia spectrum disorders (1–6). SD refers to the so-called minimal or basic self (“ipseity”), which is conceptualized as the tacit, pre-reflective level of selfhood and the ground of various aspects of conscious awareness (5). It is thought that instability of this minimal self gives rise to anomalous subjective experiences (e.g., a sense that one's thoughts are anonymous and “not mine,” a feeling as if the boundary between self and world is unclear), which may evolve into frank psychotic symptoms (7). In fact, it has been shown that SD precede the development of clinical symptoms of psychosis (8, 9) and may be observed also among patients at risk for psychosis (10–12). Koren et al. (13), in a study of non-psychotic help-seeking adolescents, showed that SD and subclinical psychotic symptoms constitute related but distinct dimensions of potential risk. Furthermore, SD has been found to be related to psychotic-like experiences (PLEs) in non-clinical samples (14–18). These studies, indicating that SD, along with PLEs, are present both in non-clinical and clinal samples, are in line with the hypothesis of a continuous distribution of psychotic symptoms in the general population (19).

Despite SD great importance to the conceptualization of psychosis, there is still insufficient empirical data on the mechanisms underlying these experiences. Recent studies have shown (16, 17) that cognitive biases, that is, dysfunctional information processing patterns leading to maladaptive conclusions and emotional dysregulation, are related to SD. Nelson et al. (20) introduced a theoretical model in which source monitoring deficits are proposed as one of the neurocognitive correlates of SD, especially in the sense of “ownership” of experiences. Source monitoring is a cognitive bias that involves difficulties in making attributions about the origins of experience, for example, whether an event happened to us, whether we just imagined it or someone told us about it. The recent study by Nelson et al. (21) confirmed the relationship between source monitoring, assessed using a variety of neurocognitive and neurophysiological tasks, and SD in patients with early psychosis. The cognitive model of positive symptoms of psychosis (22) emphasizes the importance of cognitive distortions in generating anomalous conscious experiences as well.

Another contributor to SD could be traumatic experiences. Recently, growing evidence suggests that traumatic life events play a significant role in the development of psychosis (23–25). Exposure to trauma is not only significantly more frequent in schizophrenia spectrum disorders than in the general population (25, 26), but also early adverse life events increase the frequency of PLEs in non-clinical individuals (27–29). However, the mechanisms of the relationship between trauma and psychosis still needs further investigation. Sass and Borda (30) proposed that schizophrenia spectrum disorders manifest through SD that could be *primary* or *secondary* in nature. *Primary* SD reflect disturbances in early neurodevelopment, whereas *secondary* SD appear later as *defensive-compensatory reactions* to other factors such as childhood adversities, social stress and marginalization. Haug et al. (31) found that traumatic events are indeed significantly associated with higher levels of SD in patients with schizophrenia, but only in women. Recent studies have shown that SD mediate the relationship between traumatic-life events and psychosis proneness in the general population (16, 17). These results suggested that trauma may affect the risk of psychosis through alterations in the basic sense of self.

Based on the above-mentioned literature, the aim of the current study was to test the hypothesized model of cognitive biases and exposure to traumatic life events being related to the frequency of SD which, in turn, contribute to the frequency of PLEs. Therefore, we expected an indirect effect of traumatic experiences and cognitive biases on PLEs through SD. We focused on positive PLEs, since the assumed relationships between variables of interest concern primarily this dimension of psychotic experiences. This model is an extension of one that was previously proposed and tested in a sample of university students (16). The current study was conducted amongst people drawn from the general population (i.e., a non-clinical population) who experience frequent PLEs and therefore are at psychometric risk of developing psychosis. The selected group was evaluated in terms of meeting clinical criteria of ultra-high risk (UHR) of psychosis. The goal of this strategy was to estimate the prevalence of clinical risk of psychosis among people from the general population who are not seeking help. For the measurement of cognitive biases, we used both self-report questionnaires and performance-based behavioral tasks, as they can possibly represent somewhat different constructs (32).

## Materials and Methods

### Participants

**Figure 1:**
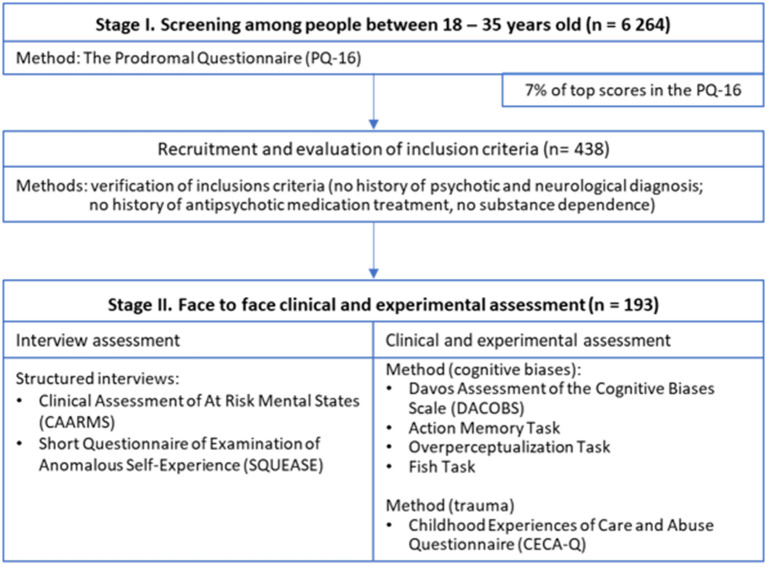
Consort flow-chart presenting the course of the study.

Our study was conducted in two stages (see Figure 1). First, a total sample of 6,264 Polish young adults (3,932 females) aged between 18 and 35 years (M = 26.51, SD = 4.76) were screened for psychometric risk of psychosis using the Prodromal Questionnaire (PQ-16) (33). Screening was carried out via Internet in collaboration with an external company specializing in acquiring for research purposes large population samples and conducting online surveys. Completing the online survey took about 20–30 min. Participants were enrolled from three large Polish cities: Warsaw (1,700,000 inhabitants), Krakow (770,000 inhabitants) and Wroclaw (640,000 inhabitants). Those who scored within 7%^1^ of top results on the PQ-16 (i.e., had frequent PLEs) and met inclusion criteria were approached to participate in the second stage of the study conducted through face to face assessment. Exclusion criteria for participants were screened with self-report questions which included: a history of any psychotic or neurological diagnosis, history of antipsychotic medication treatment and substance dependence disorder in the previous 6 months. Other psychiatric diagnoses such as major depressive disorder (without psychotic symptoms), bipolar disorder (without psychotic symptoms), personality disorders or anxiety disorders were not considered as exclusion criteria. Four hundred thirty-eight people met inclusion criteria, however 245 respondents could not be contacted or refused to participate in the second stage of the study. The final sample consisted of 193 individuals (111 females, age M = 25.36, SD = 4.69). Face to face assessment in the second stage of the study involved assessment of SD, PLEs, exposure to traumatic life events and cognitive biases. The participants' informed consent was obtained and the ethics committee of the Medical University of Warsaw approved the study.

### Measures

#### Psychotic-Like Experiences

To assess PLEs in the screening stage of the study we used the sixteen-item Prodromal Questionnaire (PQ-16) (33). The PQ-16 is a self-report questionnaire to screen for psychosis risk operationalized as a presence of PLEs. It is a shortened version of the 92-item PQ and consists of items that assess perceptual abnormalities and hallucinations, unusual thought content, delusional ideas, and paranoia as well as negative symptoms on a scale: present vs. non-present –(true vs. false) which we modified to better reflect the frequency of PLEs. Specifically, we used a four-point scale: “never”, “sometimes”, “often”, and “almost always”. The scores range from 0 to 48 points. Most of the items in the PQ-16 refer to attenuated positive psychotic symptoms. The PQ-16 has satisfactory psychometric characteristics in the assessment of PLEs with a specificity and sensitivity of 87% in discriminating patients meeting the criteria of UHR from those who do not meet UHR criteria (33). The scale was validated also in non-help-seeking populations (34, 35). We used a Polish version of the questionnaire (17). Cronbach's alpha for the total score was 0.82.

To evaluate PLEs for their clinical relevance in the second stage of the study we used the Comprehensive Assessment of At-Risk Mental States (CAARMS) (36), for the Polish version see: Jaracz et al. (37). The CAARMS is a semi-structured interview designed to investigate different aspects of attenuated psychopathology and functioning factors over time. The CAARMS consists of seven subscales: positive symptoms (subclinical delusions and hallucinations); negative symptoms (anhedonia, blunted affect, social withdrawal); cognitive changes; behavior changes; motor or physical changes; emotional disturbances; general psychopathology. This instrument allows for assessment of clinical state of UHR of psychosis. Symptoms are evaluated for their severity and frequency on scales ranging from 0 to 6. In our study, we focused on the severity and frequency of the positive symptom subscale that includes: unusual thought content, non-bizarre ideas, perceptual abnormalities, and disorganized speech. The positive symptoms subscale served as an indicator of psychosis proneness (with the combined score for the frequency and the severity subscales from 0 to 48). Cronbach's alpha for this subscale calculated in our sample was 0.82.

#### Self-Disturbances

To evaluate SD we used the SQUEASE (Møller, private materials). This is a short version of the Examination of Anomalous Self-Experience (EASE), which is a semi-structured phenomenological interview developed by Parnas et al. (38) to examine a wide variety of anomalies considered to be disorders of basic or “minimal” self. The construction of EASE was based on self-descriptions obtained from patients suffering from schizophrenia spectrum disorders. The EASE was used also in non-clinical populations (14, 39). The short version (the SQUEASE) was created by Møller, one of the co-authors of EASE. The SQUEASE consists of 13 items that are grouped into four sections: (1) Cognition and Stream of Consciousness (items include: disorder of short-term memory, attentional disturbances, ruminations-obsessions, thought interference, thought pressure, loss of thought ipseity) (2) Self-Awareness and Presence (items include: distorted first-person perspective, diminished sense of basic self, hyperrefl ectivity, derealization (3) Bodily Experiences (items include: mirror-related phenomena), (4) Existential Reorientation (items include: existential or intellectual change, feeling as if the experienced world is not truly real). These items evaluate SD for their frequency and level of presence on scales ranging from 0 to 4. The possible result for the frequency scale is in the range from 0 to 52. Cronbach's alpha calculated in our sample for the frequency scale was 0.84.

#### Exposure to Traumatic Life Events

Childhood Experiences of Care and Abuse Questionnaire (CECA.Q) (40) was used to investigate traumatic life events retrospectively such as lack of parental care (neglect and antipathy), parental psychological abuse, role reversal, parental physical abuse, and sexual abuse from an adult before the age of 17. The CECA.Q has been validated among psychotic patients (41) as well as in non-clinical samples (42, 43). It consists of different types of trauma subscales that allow for a wide assessment of traumatic life events. Cronbach's alpha for the total score in our sample was 0.96.

#### Social Functioning

Social and Occupational Functioning Assessment Scale (SOFAS) (44) is a one-item rating of an individual's functioning scored 0–100. It is intended to assess social and occupational functioning independently of the overall severity of symptoms.

#### Self-Report Cognitive Biases

The Davos Assessment of Cognitive Biases Scale (DACOBS) (45), for the Polish version, see: Gaweda et al. (46) is a self-report scale that assesses cognitive biases associated with psychosis. The questionnaire contains 42 items to be scored on a 7-point Likert scale, therefore the scores range from 42 to 294 points. All items are grouped into seven subscales and three clusters related to different types of biases: (1) specifically associated with psychosis: jumping to conclusions bias, belief inflexibility bias, attention to threat bias, external attribution bias, (2) associated with cognition: social cognition problems and subjective cognitive problems, and (3) related to coping strategies: safety behaviors. Cronbach's alpha for the total score was 0.89.

#### Performance-Based Cognitive Biases

Three computer-based tasks were used to assess different cognitive biases:

*Overperceptualization bias* was measured with a computer-based task of auditory false perceptions—Overperceptualization Task (47). The overperceptualization paradigm assesses the process by which individuals recognize auditory stimuli when in fact they are not present. In this task participants are presented with stimuli in the form of words in three conditions: (1) words can only be heard (audio condition, 60 trials), (2) words can be spoken by a lector who is heard and simultaneously seen on the screen (video condition, 60 trials), (3) before the lector appears on the screen, participants see a board with the word that will be spoken (board condition, 60 trials). Each word is accompanied by background noise making the word difficult to recognize. In 40% of stimuli in each of the three conditions, the lector does not read the word, but only moves his mouth; thus only noise can be heard. Participants have to decide after each word whether they heard a word or not and determine the degree of certainty in their decision. Subjects are instructed to respond as quickly as possible. False auditory perceptions (i.e., hearing a word when it was not spoken) serve as an indicator of overperceptualization bias (ranging from 0 to 72).

*Source monitoring deficits* were evaluated with Action Memory Task (AMT). The AMT is a computer-based task (48), for Polish version see: Gaweda et al. (49) comprising of 36 actions that are described to participants through text messages (18) or shown through images (18). Each action is imagined or performed by participants. Imagined actions are presented with a red frame, actions that have to be performed have a green frame. Each action is presented for 10 s. The memory retrieval phase starts after a short break. All imagined and performed actions are shown to the participants in random succession, as well as new ones (56 actions in total). In a recognition part of the study, participants are asked to attribute all actions as they were presented. The sum of performed actions recognized as imagined and imagined actions recognized as performed was used as an indicator of source monitoring deficits (ranging from 0 to 36).

*Jumping to conclusions bias* was measured with Fish Task (50). This task is a revised and computerized version of the beads task (51, 52) which differs from the original task in that a different scenario (lakes with fish instead of jars with beads) is presented. We used two versions of the probabilistic reasoning task which varied in terms of the discrimination ratio. The first version had a high discrimination ratio (80:20) with unambiguous evidence, whereas the second was more difficult with fish in low discriminability (60:40), representing more ambiguous evidence. The instructions were standardized and presented on a computer screen. After each fish was “caught” participants were required to make two judgments: (1) a probability judgment about the likelihood that the fish was caught from either lake A or lake B, and (2) judgment as to whether the available amount of information would justify a decision or not. The number of draws (from 1 to 10) needed to make a decision was an indicator of jumping to conclusion bias (with fewer draws indicating increased jumping to conclusions bias).

### Statistical Analysis

All statistical analyses were performed with SPSS version 25.0 and Amos version 25.0.

First, we tested for correlations among the variables of interest by calculating Pearson's correlational coefficients. SD and their relationship with exposure to traumatic life events are at the center of our interest, thus we explored this association in more detail by checking which traumatic events are related to SD. Then, we performed confirmatory factors analysis (CFA) of the latent variable of self-report cognitive biases to verify the original structure of this measure. For performance-based cognitive biases, we aimed to build a factorial model consisting of the results of three tasks: Action Memory, Overperceptualization and Fish Task. PLEs, SD and exposure to traumatic life events were represented in our model by single indicator variables. This decision was dictated by the absence of an established factorial model for positive symptoms in CAARMS, SQUEASE and CECA.Q measures. It also enabled conserving free parameters and increased stability of the parameter estimates for the models.

In the next step, we evaluated hypothesized associations with the structural equation model (SEM) in a series of path analyses to test our theoretical model. Therefore, we tested for the indirect effect of traumatic life events through SD and cognitive biases to PLEs. For this purpose, we used the bootstrap method as recommended by Preacher and Hayes (53). Due to different measurement methods (questionnaire vs. computer-based tasks) we aimed to perform path analyses separately for self-report and performance-based cognitive biases.

The goodness of fit to the data for both analyses (CFA and path analyses) were estimated with the maximum likelihood estimation procedure with the Bollen-Stine bootstrap (*n* = 2,000) procedure of correction for non-normal distribution. We verified goodness of model fit following the guidelines from literature (54): RMSEA <0.06 (The Root Mean Square Error of Approximation); SRMR <0.08 (The Standardized Root Mean Square Residual); CFI > 0.95 (Confirmatory Fit Index) and TLI > 0.95 (Tucker- Lewis Index).

## Results

### Characteristics of the Sample

The sample characteristics are presented in Table 1. Fifty-one participants from the sample of 193 individuals (26.4%) met the symptom criteria for UHR status after being interviewed with the CAARMS. However, full criteria for UHR status were not met as the group was not help-seeking and their social functioning, as measured using the SOFAS, did not meet UHR functional decline/chronic low functioning requirements.

**Table 1:** Sample characteristics.

	***N* (%)**		**Mean (SD)/Score range**
Gender		Age	25.36 (4.69)
Male	82 (42.5%)	PQ-16 (screening)	23.06 (4.49)/0–48
Female	111 (57.5%)	SQUEASE (total score)	16.12 (11.97)/0–52
Professional situation		CECA.Q	
Study	97 (50.3%)	Mother antipathy	20.33 (7.59)/8–40
Work	130 (67.4%)	Mother neglect	14.92 (6.13)/8–40
Unemployed	7 (3.6 %)	Father antipathy	21.14 (9.13)/8–40
Rent	3 (1.6%)	Father neglect	21.36 (6.91)/8–40
Education		Mother psychological abuse	18.55 (15.06)/0–85
Primary	11 (5.7%)	Father psychological abuse	16.23 (17.80)/0–85
Secondary	1 (0.5%)	Role reversal	53.58 (10.60)/17–85
Vocational	87 (45.1%)	Physical abuse	0.41 (0.49)/0–1
Incomplete higher	31 (16.1%)	Sexual abuse	0.35 (0.85)/0–3
Higher	63 (32.6%)	CAARMS (total score)	61.89 (36.18)/0–324
Psychiatric diagnosis	46 (23.8%)	Positive symptoms	9.88 (7.48)/0–48
Anxiety disorder	23 (11.9%)	SOFAS	79.71 (12.54)/0–100
Depression	30 (15.5%)	DACOBS (total score)	162.41 (27.61)/42–294
Bipolar disorder	3 (1.6%)	Jumping to conclusion	27.04 (5.11)/7–42
Obsessive-compulsive disorder	1 (0.5%)	Belief inflexibility	18.87 (5.42)/7–42
Eating disorder	4 (2.1%)	Attention to threat	27.30 (5.27)/7–42
Personality disorder	9 (4.7)	External attribution	22.45 (5.81)/7–42
Other	3 (1.6%)	Social cognition problems	26.12 (6.33)/7–42
		Subjective cognitive problems	26.26 (7.18)/7–42
		Safety behaviors	14.36 (6.17)/7–42
		Fish Task	
		JTC 80:20	5.29 (2.52)/1–10
		JTC 60:40	7.93 (2.71)/1–10
		Action Memory Task	
		Incorrect recognitions	4.06 (2.44)/0–36
		Overperceptualization Task	
		False auditory perceptions	13.59 (15.11)/0–72

*PQ-16, Prodromal Questionnaire; SQUEASE, short version of Examination of Anomalous Self-Experience; CECA.Q, Childhood Experience of Care and Abuse Questionnaire; CAARMS, Comprehensive Assessment of At-Risk Mental States; DACOBS, Davos Assessment of the Cognitive Biases Scale; Fish Task JTC, number of fish needed to make decision; Action Memory Task incorrect recognitions, number of incorrect recognition of both performed and imagined actions. The score for CAARMS is the sum of severity and frequency scales. The score for SQUEASE is the sum of frequency scale*.

### Correlational Analysis

Table 2 presents the results of the correlational analysis. The strongest significant relationship was found between SD and PLEs (*r* = 0.629, *p* < 0.001). SD correlated significantly also with self-report cognitive biases and psychological abuse from the father. Except for physical and sexual abuse, all other types of traumatic life events significantly correlated with self-report cognitive biases. It is of note that no subscale of CECA.Q was significantly related to PLEs. These statistically significant relationships among variables of interest allowed for further testing of our hypothesized model with SEM. Surprisingly, no significant relationships were found between performance-based cognitive biases and SD as well as PLEs, thus planned path analysis with these variables was not performed. Furthermore, we found a highly significant correlation between self-report cognitive biases and PLEs. Thus, we decided to investigate an additional model including this path. Gender was not included in path analyses as it was not significantly related to exposure to trauma and other variables of interest. Age significantly correlated with SD (*r* = −0.176, *p* < 0.05), PLEs (*r* = −0.181, *p* < 0.05) and cognitive biases (*r* = −0.162, *p* < 0.05). However, those paths turned out to be insignificant thus we did not include them in the final analyses.

**Table 2:** Correlational analysis.

**Variable**	**SQUEASE self-disturbance**	**CAARMS positive symptoms**	**DACOBS total score**	**Jumping to conclusion**	**Belief inflexibility**	**Attention to threat**	**External attribution**	**Social cognition problems**	**Subjective cognitive problems**	**Safety behavior**	**JTC 80:20**	**JTC 60:40**	**Action Memory Task**	**Overpercept Task**
Mother antipathy	0.043	0.016	0.265^***^	0.036	0.251^***^	0.095	0.273^***^	0.197^**^	0.160^*^	0.207^**^	−0.40	−0.031	0.010	−0.116
Mother neglect	0.051	0.025	0.202^**^	0.018	0.216^**^	0.055	0.231^**^	0.134	0.202^**^	0.064	0.013	−0.003	0.104	−0.041
Father antipathy	0.080	0.025	0.187^**^	0.090	0.102	0.095	0.258^***^	0.131	0.110	0.086	−0.037	−0.061	−0.089	−0.043
Father neglect	0.097	0.023	0.151^*^	0.026	0.002	0.093	0.239^**^	0.098	0.138	0.087	0.039	−0.029	−0.072	−0.049
Mother psychological abuse	0.127	0.038	0.210^**^	0.050	0.104	0.120	0.246^**^	0.148^*^	0.136	0.164^*^	0.074	−0.029	−0.032	−0.150^*^
Father psychological abuse	0.168^*^	0.066	0.212^**^	0.090	0.026	0.178^*^	0.250^***^	0.173^*^	0.146^*^	0.119	−0.012	−0.088	−0.081	−0.017
Role reversal	0.081	0.068	0.149^*^	0.149^*^	0.031	0.153^*^	0.159^*^	0.136	0.027	0.063	0.013	−0.07	−0.171^*^	0.028
Physical abuse	−0.012	−0.060	−0.006	0.035	0.002	−0.030	0.130	−0.046	−0.069	−0.027	0.028	−0.015	−0.077	−0.082
Sexual abuse	0.091	0.108	0.134	0.077	0.075	0.116	0.135	0.036	0.090	0.102	−0.038	−0.081	−0.104	−0.124
Self-disturbances		0.629^***^	0.275^***^	−0.095	0.154^*^	0.142^*^	0.169^*^	0.225^**^	0.354^***^	0.250^**^	−0.017	0.033	−0.069	0.032
DACOBS total	0.275^***^	0.322^***^		0.230^**^	0.700^***^	0.732^***^	0.763^***^	0.816^***^	0.708^***^	0.664^***^	−0.138	−0.093	0.155^*^	0.132
Jumping to conclusion	−0.095	−0.053	0.230^**^		0.183^*^	0.240^**^	0.128	−0.031	−0.134	−0.097	−0.118	−0.156^*^	0.027	0.059
Belief Inflexibility	0.154^*^	0.246^**^	0.700^***^	0.183^*^		0.348^***^	0.437^***^	0.518^***^	0.413^***^	0.383^***^	−0.215^**^	−0.074	0.195^**^	0.099
Attention to threat	0.142^*^	0.144^*^	0.732^***^	0.240^**^	0.348^***^		0.513^***^	0.528^***^	0.365^***^	0.466^***^	−0.085	−0.122	0.096	0.081
External attribution	0.169^*^	0.174^*^	0.763^***^	0.128	0.437^***^	0.513^***^		0.585^***^	0.481^***^	0.386^***^	−0.057	−0.044	0.221^**^	0.087
Social cognition problems	0.225^**^	0.321^***^	0.816^***^	−0.031	0.518^***^	0.528^***^	0.585^***^		0.596^***^	0.501^***^	−0.179^*^	−0.082	0.002	0.155^*^
Subjective cognitive problems	0.354^***^	0.295^***^	0.708^***^	−0.134	0.413^***^	0.365^***^	0.481^***^	0.596^***^		0.377^***^	0.048	0.073	0.036	0.034
Safety behavior	0.250^**^	0.311^***^	0.664^***^	−0.097	0.383^***^	0.466^***^	0.386^***^	0.501^***^	0.377^***^		−0.075	−0.077	0.118	0.110
CAARMS positive symptoms	0.629^***^		0.322^***^	−0.053	0.246^**^	0.144^*^	0.174^*^	0.321^***^	0.295^***^	0.311^***^	0.002	0.064	−0.056	0.092
JTC 80:20	−0.017	0.002	−0.138	−0.118	−0.215^**^	−0.085	−0.057	−0.179^*^	0.048	−0.075		0.463^***^	0.010	−0.106
JTC 60:40	0.033	0.064	−0.093	−0.156^*^	−0.074	−0.122	−0.044	−0.082	0.073	−0.077	0.463^***^		0.058	−0.071
Action Memory Task	−0.069	−0.056	0.155^*^	0.027	0.195^**^	0.096	0.221^**^	0.002	0.036	0.118	0.010	0.058		0.123
Overpercept Task	0.032	0.092	0.132	0.059	0.099	0.081	0.087	0.155^*^	0.034	0.110	−0.106	−0.071	0.123	

* p < 0.05,

** p < 0.01,

*** p < 0.001.

*SQUEASE, short version of Examination of Anomalous Self-Experience; CAARMS, Comprehensive Assessment of At-Risk Mental States; DACOBS, Davos Assessment of the Cognitive Biases Scale; JTC, Jumping to Conclusion Fish Task*.

### Measurement Model

Due to the inability to confirm the original latent structure of the 42-item DACOBS questionnaire measuring cognitive biases, we decided to use as indicator variables the sum of the points obtained in each subscale instead of all single items. We removed only jumping to conclusion subscale because of its insignificant loading. Thus, the final latent structure for self-report cognitive biases consisted of six indicators (belief inflexibility, attention to threat, external attribution, social cognition problems, subjective cognitive problems, and safety behaviors) and fit the data well [χ2 (7) = 2.791, *p* > 0.05; RMSEA = 0.00 (90% CI = 0.000–0.037), CFI = 1.00, TLI = 1.023, SRMR = 0.014]. For PLEs, SD and traumatic life events, we used single indicator variables, which was the sum of the frequency and severity scales obtained for all items in the positive symptoms subscale of CAARMS and in father psychological abuse subscale of CECA.Q. In the case of SD we used only the sum of the frequency scale, as the level of presence is a qualitative scale.

### Path Analyses

Results of first path analysis suggested a model that fit the data well [χ2 (23) = 33.780, *p* = 0.068; RMSEA = 0.049 (90% CI = 0.000–0.083), CFI = 0.980, TLI = 0.968, SRMR = 0.044]. However, the path from father psychological abuse to SD turned out to be insignificant. A detailed model is presented in the Supplementary Figure 1. Therefore, we checked for correlations between SD and all single items representing trauma. We selected 12 specific items measuring trauma that were significantly related to SD and used their sum as an indicator variable of exposure to trauma in further path analyses. Those items originally constituted psychological abuse (nine items), role reversal (two items) and parental care (one item) subscales. Detailed correlational analysis between the SQUEASE and CECA.Q items is presented in Supplementary Table 1.

**Figure 2:**
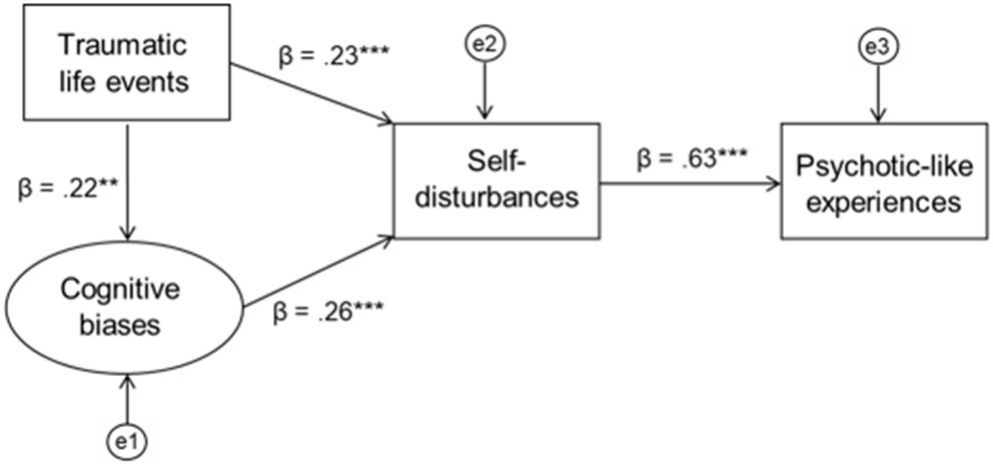
The model of the direct relationship between self-disturbances and psychotic-like experiences with childhood trauma and self-report cognitive biases as the potential contributors to self-disturbances.

The first model with initially hypothesized relationships is depicted with its standardized path coefficients (standardized regression weights) in Figure 2. The bootstrapping estimate revealed a significant standardized indirect effect of traumatic life events through SD and cognitive biases to PLEs (β = 0.181, 95% CI = 0.102–0.267, *p* = 0.001). This model explained 39.6% of the variance in PLEs. All of the model fit indices were satisfactory: χ2(24) = 40.847; *p* = 0.017; RMSEA = 0.060 (90% CI = 0.025–0.091), CFI = 0.968, SRMR = 0.059, TLI = 0.953.

**Figure 3:**
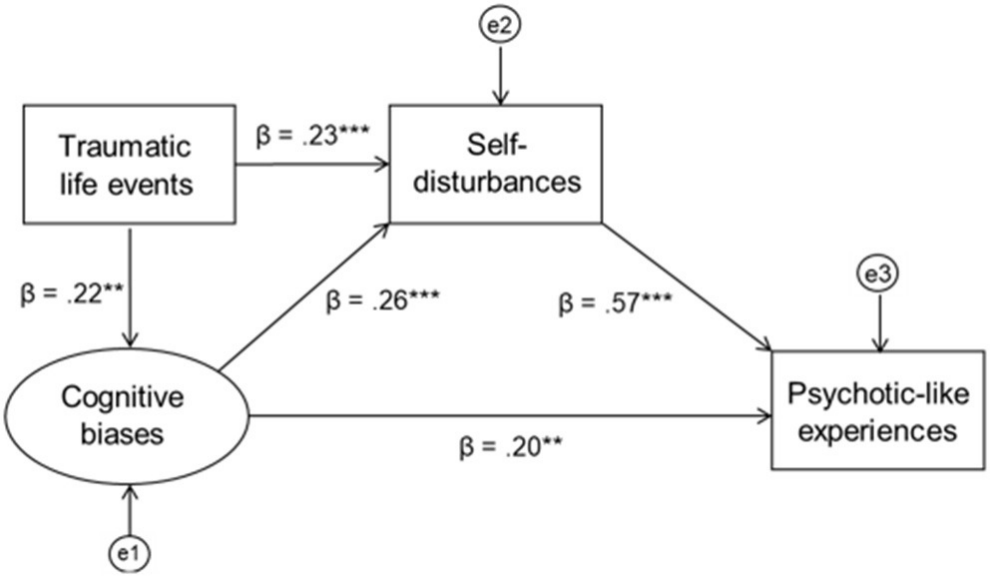
The model of the indirect relationship between childhood trauma and psychotic-like experiences with self-disturbances and cognitive biases as the potential mechanisms underlying this relationship.

The second model includes an additional path from self-report cognitive biases to PLEs and is presented in Figure 3. Standardized indirect effect of traumatic life events through SD and cognitive biases to PLEs was significant (β = 0.207, 95% CI = 0.126–0.293, *p* = 0.001). The percentage of the variance explained in PLEs was equal to 43.1%. The model has a good fit: χ2(23) = 31.201; *p* = 0.118; RMSEA = 0.043 (90% CI = 0.00–0.078), CFI = 0.985, SRMR = 0.041, TLI = 0.976.

## Discussion

In the current study, we focused on the relationship between SD and PLEs with exposure to traumatic life events and cognitive biases as potential mechanisms underlying SD. All of the hypothesized associations were found to be statistically significant and the model fit the data well. SD along with its postulated mechanisms explained a substantial part of the variance in PLEs, pointing to the importance of this construct in elucidating and understanding psychosis risk. Results of our study, although obtained in a non-clinical sample, are in line with the basic-self-disorder model of schizophrenia (5, 55).

Several points should be noted regarding the results of our study. First, we found a statistically significant indirect effect of trauma on PLEs, which is consistent with many theoretical and empirical accounts on the role of trauma in shaping psychosis risk (56–59). However, the strength of this relationship was smaller than we expected. One of the possible reasons could lie in the nature of our sample. Although participants in the study reported the highest frequency of PLEs from the screening sample, they functioned well-socially and professionally. In fact, one-quarter of them met the symptomatic criteria for UHR and it was a relatively high level of their functioning that excluded a full diagnosis of this kind. It is likely that individuals with higher social and professional functioning have been less frequently exposed to traumatic-life events (60).

Moreover, we did not find a direct relationship between trauma and PLEs, which contradicts the results obtained in other research (61–63). However, de Vos et al. (64) in their recent study among UHR for psychosis youth acquired similar outcomes, that is, childhood trauma appeared to be unrelated to attenuated psychotic symptoms. In fact, some researchers found the relationship between maltreatment and PLEs to be fully mediated by various mechanisms such as borderline personality features, dissociation, perceived stress, negative-other beliefs or external locus of control (16, 65–67). Those results are consistent with the postulate that trauma alone is not a sufficient factor to cause PLEs (68). The results of our study suggest that to provoke PLEs exposure to trauma first may need to disturb the basic sense of self and trigger dysfunctional changes in information processing from the environment. According to Sass and Borda (30) the relationship between trauma and SD could be explained by dissociative reactions. They introduced the concept of *secondary* diminished self-presence, one of the aspects of SD, that could be the result of defensive—and in this sense secondary—dissociative reactions to traumatic situations (55). Indeed, the associations between trauma and SD or trauma and dissociation were found in both clinical (31, 69) and non-clinical (16, 17, 66) samples. It has also been shown that dissociative processes are related to childhood adversity in patients with schizophrenia spectrum disorders (69, 70) and in psychosis proneness (71).

The role of the second possible mechanism of SD—cognitive biases—is somewhat more difficult to interpret. Although self-report cognitive distortions showed an association between trauma and PLEs, this was not the case for *performance*-based cognitive biases. None of the tasks we used in our study was significantly related to neither SD nor PLEs. Moreover, even the correlations between the two distinct measures of cognitive biases turned out to be much smaller and less numerous than we expected. It is possible that self-report and behavioral tasks assess two different aspects of cognitive biases, that is, the first may capture subjective opinion and be a more or less stable, trait-like construct, whereas the second is the objective measure of distortions that are present here and now (i.e., more state like) and in relation to specific perceptual material. Therefore, performance in behavioral tasks may be more influenced by immediate context and affective state for example, whether the person is feeling stressed, relaxed, distracted at the time of testing. This discrepancy between self-report measures and objective neuropsychological results has been observed in previous studies (72, 73). Another possible reason is that behavioral tasks could be less sensitive measures for capturing biased cognitive processes in non-clinical samples. Future studies should investigate the relationship between objective and subjective measures of information- processing biases in more detail and in clinical groups.

It is worth noting that although we hypothesized cognitive biases affect PLEs solely through SD, correlation analysis clearly indicated a highly significant direct relationship between cognitive biases and PLEs. Path analyses suggest that although there is an indirect effect of trauma on PLEs, our results suggest cognitive biases also make a direct and unique contribution to PLEs that goes beyond the presence of SD. This is in line with a cognitive model of psychosis (22, 74) which assumes that biased information processing can directly give rise to psychotic symptoms. Indeed, previous studies have shown that delusions or delusional ideation, for example, are associated with attributional biases (75, 76) or an exaggerated tendency to pay attention to threat (77–79). It has been postulated that exposure to traumatic events in childhood distorts cognitive schemas in a way that people view the world as threatening and attribute negative events and experiences to external factors (17, 29, 66, 80). These distorted cognitive schemas are then used to interpret and explain new experiences in a paranoid framework (56).

Our model may have clinical implications. Different risk factors such as a history of exposure to trauma, cognitive biases and SD should be considered jointly in screening procedures to maximize chances for identifying people who are at the highest risk for psychosis. As SD was the variable that had the highest regression coefficient with PLEs, particular attention should be paid to identify these anomalous self-experiences when detecting individuals at risk and preventing the development of full-blown psychosis (8). Cognitive biases that were found in our study to be both directly and indirectly associated with PLEs can be successfully addressed in cognitive-behavioral therapy (CBT), for example through metacognitive training (81, 82). Furthermore, Škodlar et al. (83) provided a compelling theoretical account of how psychotherapy may be targeted to the amelioration of SD.

The results of the study should be interpreted in light of its strengths and limitations. The strengths of the study lie in the combination of different levels of measures, namely self-report, clinical interviews and behavioral tasks. To the best of our knowledge, this is the first study simultaneously examining trauma exposure and different types of cognitive biases and their relation to SD and PLEs in a non-clinical sample. However, the cross-sectional design of the study precludes causal inference. Therefore, future longitudinal studies in clinical samples are needed to address causality and capture the change in SD and PLEs over time. Further validation of our model should be carried out using the full version for the clinical interview of SD, the EASE (38). This would allow for an examination of relationships between specific aspects of SD and other variables of interest. Moreover, it should be noted that we focused only on positive PLEs, therefore our results do not relate to the entire range of PLEs, such as negative or disorganized PLEs. Lastly, as our model was tested in a specific sample of people with frequent PLEs, thus the results should not be generalized to the clinical risk of psychosis or people with low or medium frequency of PLEs.

## Data Availability Statement

The raw data supporting the conclusions of this article will be made available by the authors, without undue reservation.

## Ethics Statement

The studies involving human participants were reviewed and approved by the ethics committee of the Medical University of Warsaw, Poland. The patients/participants provided their written informed consent to participate in this study.

## Author Contributions

ŁG and RP-U: conceptualization and writing. RP-U: data curation, formal analysis, roles/writing—original draft, and visualization. ŁG: funding acquisition. RP-U, AC, DF, and ŁG: investigation. ŁG, DF, and AC: methodology, project administration, resources, and software. LG: supervision. All authors review & editing.

## Conflict of Interest

The authors declare that the research was conducted in the absence of any commercial or financial relationships that could be construed as a potential conflict of interest.

## Footnotes

^1^We planned to recruit approximately 200 participants from approximately 6000 subjects (3.3% of the sample studied) who would achieve scores on the PQ-16 within the top 10%. We chose a wider percentage of the highest scores to recruit from, expecting that not all participants would meet the inclusion criteria and would be willing to take part in the second stage of the study. Finally, we examined 193 people whose results on the PQ-16 against the entire sample turned out to be in the top 7%.
