# Supplementary material for: The Indirect Effect of Trauma via Cognitive Biases and Self-Disturbances on Psychotic-Like Experiences
Source: Front Psychiatry. 2021 Mar 29;12:611069. doi: 10.3389/fpsyt.2021.611069 (PMC8039125; doi:10.3389/fpsyt.2021.611069)
Supplement: Supplementary file 1 [file Data_Sheet_1.docx]

**SUPPLEMENTARY MATERIALS**

**Table 1.** Correlational analysis between CECA.Q items and SQUEASE total score.

|  | **SQUEASE** |
| --- | --- |
| **CECA.Q subscales’ items:** |  |
| ***Mother antipathy and neglect (2A)*** |  |
| 1. | -.035 |
| 2. | -.024 |
| 3. | .022 |
| 4. | .059 |
| 5. | .032 |
| 6. | -.125 |
| 7. | .095 |
| 8. | .086 |
| 9. | .115 |
| 10. | .125 |
| 11. | .017 |
| 12. | -.033 |
| 13. | .013 |
| 14. | .092 |
| 15. | .078 |
| 16. | -.013 |
| ***Mother psychological abuse (2B)*** |  |
| 1. | .199** |
| 2. | .074 |
| 3. | .153* |
| 4. | .185* |
| 5. | .056 |
| 6. | -.069 |
| 7. | .030 |
| 8. | .164* |
| 9. | -.062 |
| 10. | .053 |
| 11. | .069 |
| 12. | .044 |
| 13. | .000 |
| 14. | .061 |
| 15. | -.019 |
| 16. | .025 |
| 17. | -.074 |
| ***Father antipathy and neglect (3A)*** |  |
| 1. | .029 |
| 2. | .156* |
| 3. | .094 |
| 4. | .081 |
| 5. | .035 |
| 6. | .007 |
| 7. | .056 |
| 8. | .063 |
| 9. | .131 |
| 10. | .074 |
| 11. | .048 |
| 12. | .019 |
| 13. | .060 |
| 14. | .092 |
| 15. | .066 |
| 16. | .050 |
| ***Father psychological abuse (3B)*** |  |
| 1. | .196** |
| 2. | .034 |
| 3. | .202** |
| 4. | .225** |
| 5. | .051 |
| 6. | .036 |
| 7. | .113 |
| 8. | .201** |
| 9. | .171* |
| 10. | .091 |
| 11. | .010 |
| 12. | .015 |
| 13. | .082 |
| 14. | .031 |
| 15. | .099 |
| 16. | -.102 |
| 17. | .060 |
| ***Role reversal (3C)*** |  |
| 1. | -.082 |
| 2. | -.064 |
| 3. | .026 |
| 4. | -.065 |
| 5. | .118 |
| 6. | .040 |
| 7. | .123 |
| 8. | .086 |
| 9. | .017 |
| 10. | .143* |
| 11. | .026 |
| 12. | .136 |
| 13. | .054 |
| 14. | -.065 |
| 15. | -.089 |
| 16. | .146* |
| 17. | .070 |
| ***Physical abuse (5)*** |  |
| 1. | -.012 |
| ***Sexual abuse (6)*** |  |
| 1. | .047 |
| 2. | .097 |
| 3. | .102 |

Note: * p < 0.05, ** p < 0.01

**Fig. 1.** Path analysis of the relationships among self-disturbances, self-report cognitive biases, father psychological abuse and psychotic-like experiences.

**
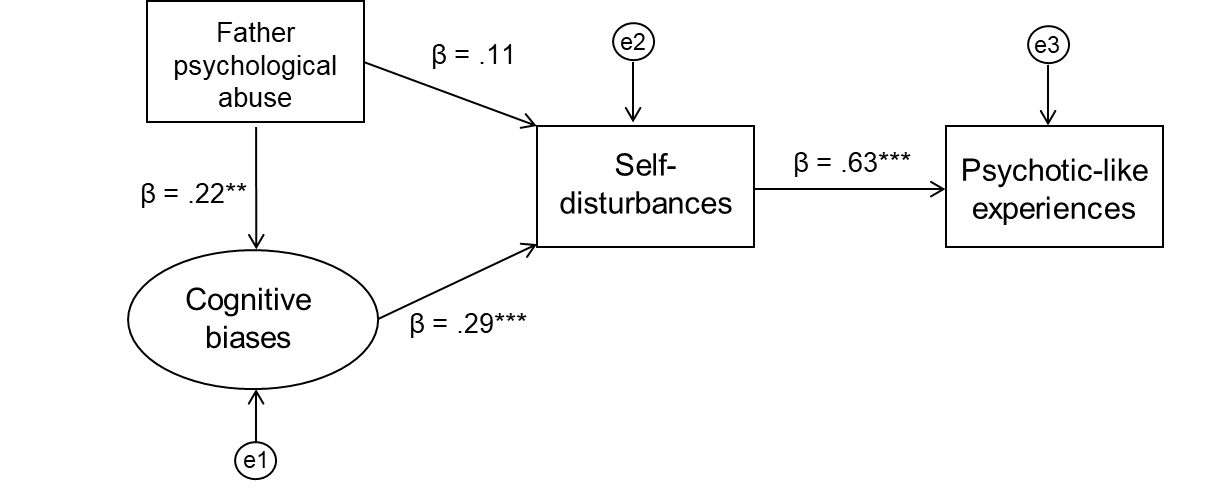
**
